# The association between internet addiction and psychiatric co-morbidity: a meta-analysis

**DOI:** 10.1186/1471-244X-14-183

**Published:** 2014-06-20

**Authors:** Roger C Ho, Melvyn WB Zhang, Tammy Y Tsang, Anastasia H Toh, Fang Pan, Yanxia Lu, Cecilia Cheng, Paul S Yip, Lawrence T Lam, Ching-Man Lai, Hiroko Watanabe, Kwok-Kei Mak

**Affiliations:** 1 Department of Psychological Medicine, Yong Loo Lin School of Medicine, National University of Singapore, Singapore, Singapore; 2 Department of Medical Psychology, Shandong University, Shandong, China; 3 Department of Psychology, University of Hong Kong, Hong Kong; 4 Department of Social Work and Social Administration, University of Hong Kong, Hong Kong; 5 Discipline of Pediatrics and Child Health, Sydney Medical School, The University of Sydney, Sydney, Australia; 6 Department of Psychology, Chinese University of Hong Kong, Hong Kong; 7 Department of Children and Women’s Health, Osaka University Graduate School of Medicine, Osaka, Japan; 8 Department of Community Medicine and School of Public Health, Faculty of Medicine, University of Hong Kong, 21 Sassoon Road, Pokfulam, Hong Kong

**Keywords:** Internet addiction, Depression, Anxiety, Alcohol abuse, Attention deficit, Hyperactivity

## Abstract

**Background:**

This study evaluates the association between Internal Addiction (IA) and psychiatric co-morbidity in the literature.

**Methods:**

Meta-analyses were conducted on cross-sectional, case–control and cohort studies which examined the relationship between IA and psychiatric co-morbidity. Selected studies were extracted from major online databases. The inclusion criteria are as follows: 1) studies conducted on human subjects; 2) IA and psychiatric co-morbidity were assessed by standardised questionnaires; and 3) availability of adequate information to calculate the effect size. Random-effects models were used to calculate the aggregate prevalence and the pooled odds ratios (OR).

**Results:**

Eight studies comprising 1641 patients suffering from IA and 11210 controls were included. Our analyses demonstrated a significant and positive association between IA and alcohol abuse (OR = 3.05, 95% CI = 2.14-4.37, z = 6.12, P < 0.001), attention deficit and hyperactivity (OR = 2.85, 95% CI = 2.15-3.77, z = 7.27, P < 0.001), depression (OR = 2.77, 95% CI = 2.04-3.75, z = 6.55, P < 0.001) and anxiety (OR = 2.70, 95% CI = 1.46-4.97, z = 3.18, P = 0.001).

**Conclusions:**

IA is significantly associated with alcohol abuse, attention deficit and hyperactivity, depression and anxiety.

## Background

Internet addiction (IA) was initially considered a new psychiatric disorder in the fifth edition of the Diagnostic and Statistical Manual of Mental Disorders (DSM-5) [1]. Following publication of the DSM-5 in 2013 however, internet gaming disorder was instead, contemplated [2]. The proposed diagnostic criteria for internet gaming disorder mirror the core criteria of substance misuse disorders, and include the following: 1) preoccupation with internet gaming; 2) occurrence of withdrawal symptoms when internet gaming access is removed; 3) the need to spend increasing amounts of time on internet gaming; 4) unsuccessful attempts to control internet gaming; 5) continued excessive internet gaming despite negative psychosocial consequences; 6) loss of previous interests hobbies and entertainment as a result of excessive internet gaming; 7) the use of internet gaming to relieve dysphoria; 8) deceiving others about internet gaming; and 9) loss of relationship, educational opportunity or career as a result of internet gaming. IA is a broader construct which comprises internet gaming and other forms of addictive internet usage (e.g. addictive downloading, excessive use of social networking sites and addictive online shopping). As a result, the construct of IA warrants empirical attention for further consideration as a formal psychiatric disorder in the prospective diagnostic nomenclature.

With the growth of internet users worldwide, IA has become a pandemic in the new era [1]. Ko et al. [3] reported that the prevalence of IA ranged from 1% to 36.7%. The variability in prevalence rates across the studies that were reviewed may be attributed to variations in accessibility of the internet in different countries, definitions of IA and diagnostic instruments [4]. IA and psychiatric co-morbidity may co-occur as a dual diagnosis and engender significant impact on patients and existing treatment services. There is a pressing need to assess the strength of the association between IA and psychiatric co-morbidity. Excessive internet use was found to be associated with psychiatric conditions such as depression [5], insomnia [6], attention deficit and, hyperactivity and social phobia [7]. Ko et al. [3] published a review of the literature and reported that IA was associated with attention deficit and hyperactivity disorder (ADHD), major depressive disorder and social anxiety disorder. It was however, a descriptive review which lacked statistical analyses to support such associations. Conversely, a meta-analysis is able to estimate the aggregate prevalence and odds ratio (OR) of psychiatric co-morbidity in patients suffering from IA as compared to healthy controls. Carli et al. [8] published a systematic review on the association between pathological internet use and combined psychopathology. According to the review, the prevalence rates of psychiatric co-morbidity varied from 57% with symptoms of anxiety to 100% with attention deficit and hyperactivity. The high prevalence rate reported in this review has to be interpreted with caution because the authors did not specify the statistical method that was used to calculate the aggregate prevalence. Furthermore, the pooled OR was not calculated for each psychiatric co-morbidity. Carli et al. also concluded that depression and ADHD demonstrated the strongest correlations with pathological internet use. However, these correlations need to be corroborated by a methodologically robust meta-analysis. The amalgam of studies on IA and psychiatric co-morbidity seemed to elucidate heterogeneity yet both reviews did not report the level of heterogeneity. The objective of this meta-analysis was to evaluate the association between IA and psychiatric co-morbidity. We performed a meta-analysis of cross-sectional, case-control and cohort studies to determine the overall strength of the putative association between IA and psychiatric co-morbidity. We also computed the aggregate prevalence of psychiatric co-morbidity among people with IA as compared to controls without IA, and reported the level of heterogeneity for each psychiatric co-morbidity. As IA is a confluence of substance use and addictive disorder, we hypothesized that the prevalence of psychiatric co-morbidity in IA would be similar to the prevalence of psychiatry co-morbidity in substance use and addictive disorders in general.

## Methods

Online databases were searched from inception to June 2012: PubMed (from 1966), Embase (from 1980), PsychINFO (From 1806), BIOSIS (from 1926), Science Direct (from 2006) and Cochrane CENTRAL (from 1993). The search terms used were permutations of keywords for internet addiction (internet addiction, problematic internet use) and psychiatric co-morbidity (depress*, mood, bipolar, ADHD, attention deficit hyperactivity, conduct, anxiety, phobia, panic, psychosis, schizo*, eating, anorexia, bulimia, personality, antisocial, borderline, narcissistic, histrionic, schizoid, schizotypal, paranoid, dependent, anxious, avoidance, obsessive compulsive, anankastic, histrionic, alcohol, cannabis, marijuana, amphetamine, cocaine, stimulant, ecstasy, hallucinogen, ketamine, phencyclidine and heroin) where the symbol* indicates truncation. Abstracts presented in major international conferences and unpublished dissertations were manually searched, and the authors of selected correspondences were contacted for further information. References from the retrieved papers were hand-searched.

### Inclusion criteria

We sought all cross-sectional, case-control and cohort studies that examined the relationship between IA and psychiatric co-morbidity. Studies were included if they 1) were human studies involving patients suffering from IA and healthy controls without IA; 2) adopted a formal definition of IA based on the Young’s Internet Addiction Test, Chen Internet Addiction Scale or other well-defined criteria. Young’s Internet Addiction Test defines IA predominantly by 1) withdrawal; 2) social problems; 3) time management and performance; and 4) reality substitute [9]. Chen Internet Addiction Scale defines IA mainly by 1) withdrawal, compulsive use, and tolerance; 2) interpersonal and health-related problems; and 3) time management problems [10]; 3) analyzed psychiatric co-morbidity as the main variable of interest or as a covariate; 4) analyzed psychiatric co-morbidity as the dependent variable where the psychiatric co-morbidity was assessed by standard questionnaires; and 5) provided sufficient information to calculate the aggregate prevalence of psychiatric co-morbidity in the IA group and the control group. For case-control studies to be included, the studies must have drawn some comparisons regarding the presence of a psychiatric co-morbidity between the IA group and the control group. For inclusion of prospective studies in the meta-analysis, sufficient information was required to estimate the aggregate prevalence and pooled OR.

### Exclusion criteria

Studies were excluded if they 1) did not provide sufficient information to calculate the aggregate prevalence and OR; 2) did not provide a specific definition of, and criteria for IA; and 3) the authors did not respond to provide further information upon request including the psychiatric co-morbidity directly related to internet use (e.g. online gambling). Articles with abstracts that were written in the English or Chinese language but had full texts written in non-English or non-Chinese languages were excluded (Studies published in Chinese and Korean which were excluded and the reasons for exclusion are found in Additional files 1 and 2). In addition, we excluded case reports, case series and studies only reported results on electroencephalogram (EEG), event-related potentials, imaging, intervention and other phenomenon such as decision making, lifestyle, impulsivity and sexual attitude without reporting psychiatric co-morbidity.

### Selection of articles

All articles were anonymised (i.e. blinded title, author(s), year of publication and journal name) prior to selection. Selection of relevant publications was conducted independently by two authors (i.e. TYT and MWZ). Articles were initially screened on the basis of titles and abstracts. The short-listed articles were then evaluated on study design, and screened for the inclusion and exclusion criteria listed above. Disagreement regarding the inclusion or exclusion of retrieved papers was resolved by discussion with the first author (i.e. RCH) before the list of articles to be used in the meta-analysis was formalised. There were 13 studies published in Chinese (please see Additional file 1). None of these studies reported psychiatric co-morbidity including depression, anxiety, ADHD and alcohol abuse. There were 10 studies published in the Korean language of which 8 studies did not meet the inclusion criteria because psychiatric co-morbidity was not reported (please see Additional file 2). One study reported the mean depression scores but not the number of participants suffering from depression. One study reported alcohol use (i.e. 1-2 times per week) but not alcohol abuse. As a result, these two studies were excluded. All procedures were in accordance with the guidelines for the meta-analysis of observational studies (MOOSE) in epidemiology [11].

### Statistical methods

#### Data extraction

The following information was extracted from each article, crossed-checked by the second and last author, and recorded on a standardized data collection form: a) publication details (family name of the first author, and other citation details including geographic locale and year of publication); b) the number of IA patients and healthy controls; c) the number of IA cases and controls for each psychiatric comorbidity; d) source of healthy controls; e) descriptions of instruments for assessment of psychiatric co-morbidity; and f) demographics of participants including mean age, proportion of gender, and proportion of ethnicities.

#### Statistical analysis

**Figure 1:**
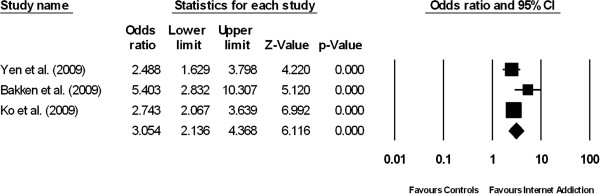
Forest plot comparing the odds ratios of having co-morbid alcohol abuse among patients suffering from internet addiction versus controls without internet addiction.

**Figure 2:**
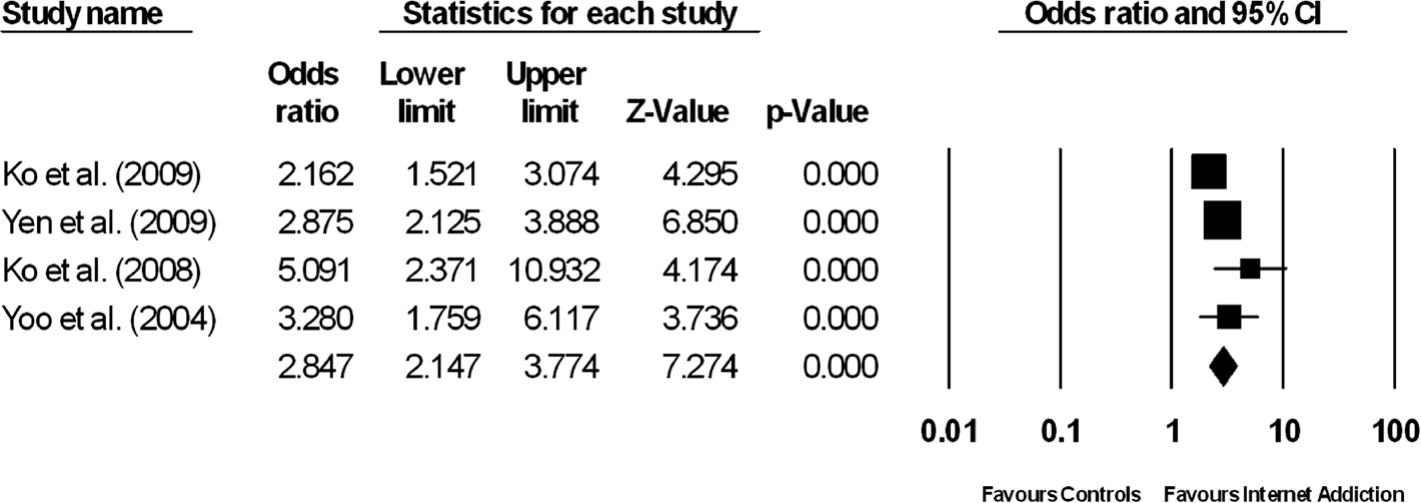
Forest plot comparing the odds ratios of having co-morbid attention deficit hyperactivity among patients suffering from internet addiction versus controls without internet addiction.

**Figure 3:**
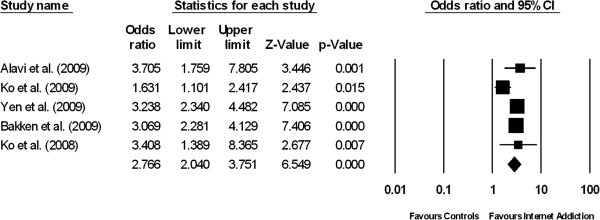
Forest plot comparing the odds ratios of having co-morbid depression among patients suffering from internet addiction versus controls without internet addiction.

**Figure 4:**
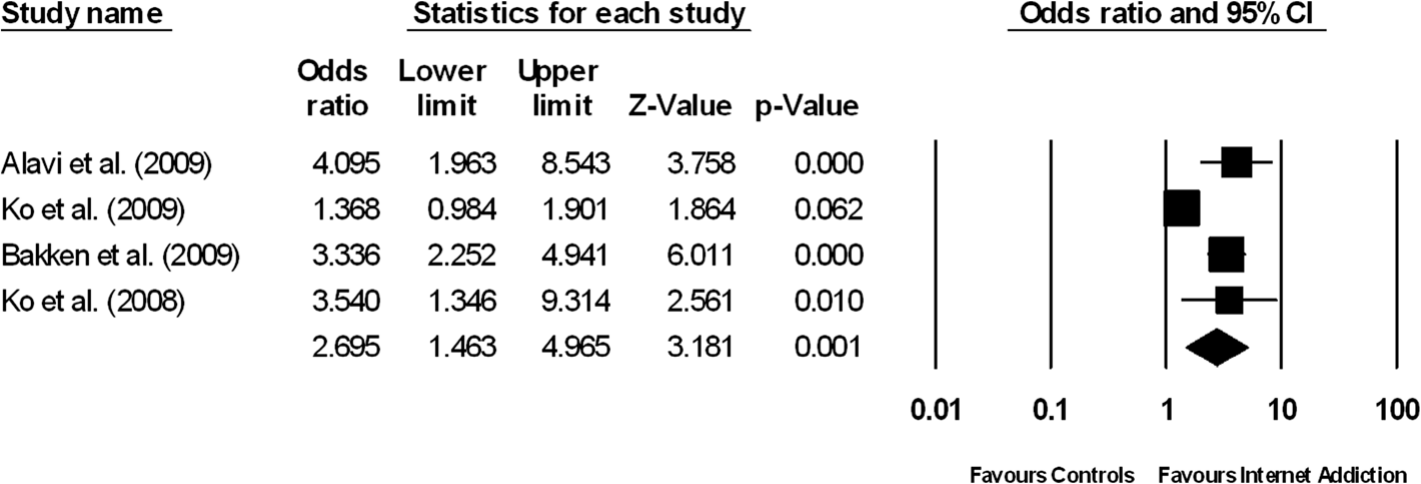
Forest plot comparing the odds ratios of having co-morbid anxiety among patients suffering from internet addiction versus controls without internet addiction.

All statistical analyses were performed with Comprehensive Meta-Analysis Version 2.0 using the random–effects models for aggregate prevalence and pooled OR. The random-effect model was used because it assumes varying effect size between studies but leads to a markedly conservative null-hypothesis model [12], and it takes into consideration subject-specific effects [13-15]. All studies reported the presence and absence of a psychiatric comorbidity as dichotomous outcomes. Thus, summary statistics including the aggregate prevalence of a psychiatric comorbidity, 95% CI, Q-value, degree of freedom, p-value and tau^2^ were reported for the IA group and the healthy controls. To test the hypothesis that a specific psychiatric comorbidity was in fact more common in the IA group, the pooled OR was calculated. The pooled OR, 95% CI, z-value and P-value were reported for each figure of psychiatric comorbidity. Significant statistical difference was set at P < 0.05 for all analyses. Between-study heterogeneity was assessed with the I^*2*^ statistic, which describes the percentage of variability among effect estimates beyond that expected by chance [16]. As a reference, I^*2*^ values of 25% was considered low, 50% moderate, and 75% high. Egger’s regression tests were performed to detect publication bias. If significant publication bias was present, the classic fail-safe test would be performed to determine the number of missing studies required for the P-value of publication bias among the observed studies to approximate > 0.05. Forest plots (Figures 1, 2, 3 and 4) for each of the co-morbidity of IA were generated to summarize individual study estimates and the overall estimate, and to compare the odds ratio between the IA and normal groups.

#### Subgroup analysis

We undertook a subgroup analysis to investigate the effects of age on the prevalence of psychiatric co-morbidity between the IA and control groups. We compared the prevalence of psychiatric co-morbidity among three subgroups: 1) adolescents (10-18 years of age); 2) young adults (19-39 years of age); and 3) middle-aged adults and the elderly (40-76 years of age). The age ranges of the aforementioned groups were determined by the mean age and overall age range of the subjects recruited.

## Results

**Figure 5:**
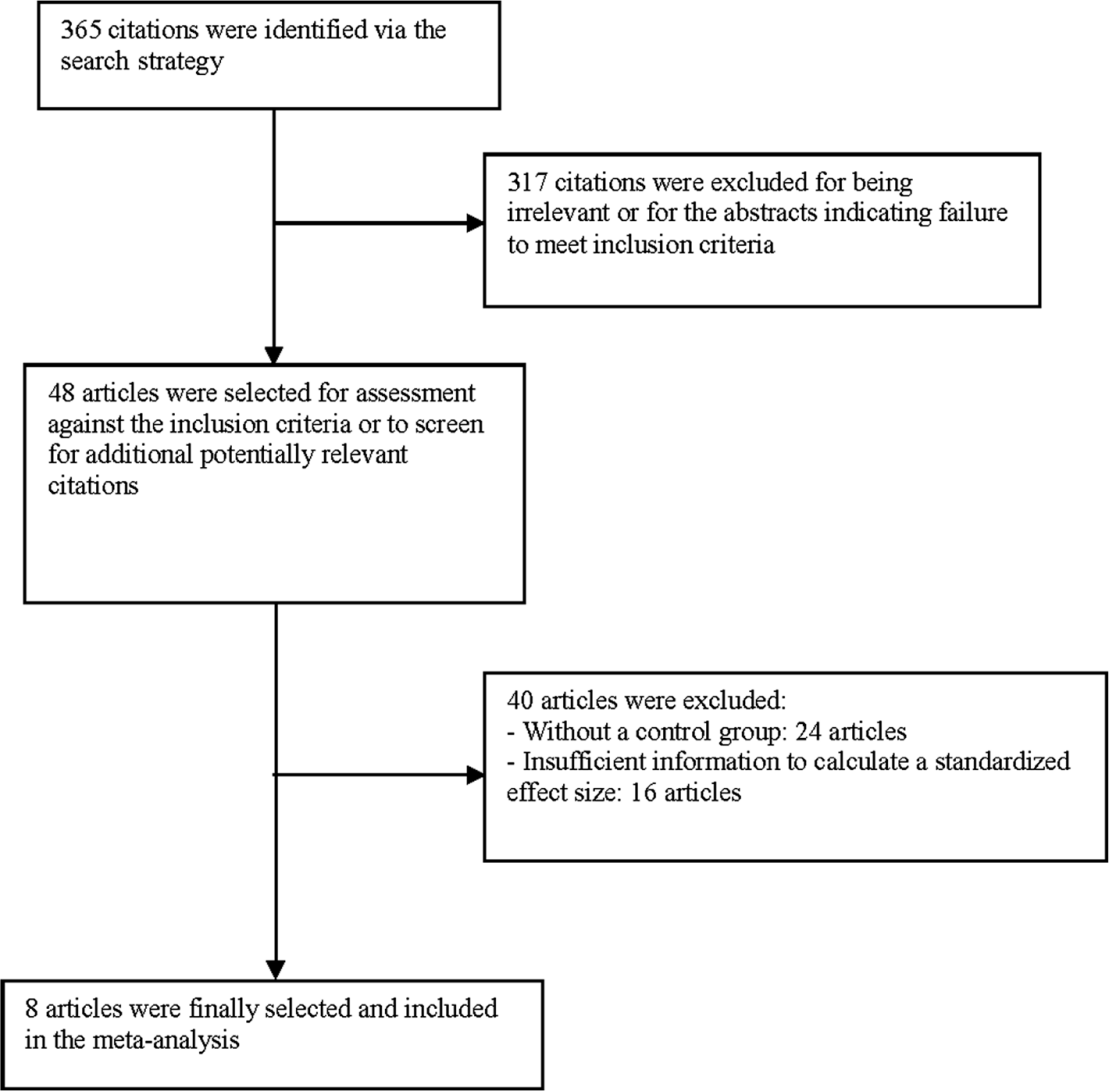
Literature search profile and study selection process.

Eight studies comprising a total of 1641 patients with IA and 11210 controls without IA were included in the analysis (Figure 5). The studies consisted of patients mainly from Asian countries. Descriptive characteristics of the study populations are shown in Table 1. Subgroup analyses are shown in Table 2.

**Table 1:** Descriptive characteristics of studies between internet addiction and psychiatric co-morbidity

**Study (date)**	**Study location**	**Study design**	**Population**	**Case definition**	**Description of controls**	**Number of subjects (n) (IA/controls)**	**Proportion of gender in all subjects (% males)**	**Proportion of gender in IA patients (% males)**	**Mean age of all subjects (in years)**	**Method of assessment for psychiatric co-morbidity**	**Psychiatric comorbidity studied**	**Prevalence of psychiatric co-morbidity (IA/ controls)**
Alavi et al. (2011) [17]	Iran	Cross- sectional	College student	YDQ and IAT	Community sample	36/214	38.0	61.1	22.5	SCL-90-R	Depression	44.4/17.8
											Anxiety	50.0/19.8
Ko et al. (2009) [18]	Taiwan	Observational cohort study	7^th^ grade students from 10 junior high schools	CIAS	Community sample	276/1572	56.1	63.8	12.4	CES-D	Depression	14.3/9.3
										ADHDA	ADHD	19.5/10.1
										BV-FNE	Anxiety	20.3/15.7
Yen et al. (2009) [19]	Taiwan	Case -control	College students	CIAS	Community sample	246/1746	29.2	45.1	20.5	CES-D	Depression	26.4/10.1
										AUDIT	Alcohol	13.0/5.7
Yen et al. (2009) [20]	Taiwan	Case control	Students from 8 colleges	CIAS	Community sample	338/2281	33.5	51.8	20.5	ASRS	ADHD	20.7/8.3
Bakken et al. (2009) [21]	Norway	Cross- sectional	National postal survey	YDQ score of ≥ 3	Community sample and YDQ score of 2 and below	212/3181	47.1	NA	45.8	Self-reported feelings of depression, anxiety, alcohol abuse	Depression	36.0/15.4
											Anxiety	16.5/5.6
											Alcohol	6.1/1.2
Ko et al. (2008) [22]	Taiwan	Observational cohort study	Student from 2 classes per grade from a random selection of 3 senior high schools and 7 vocational high school	CIAS	Community sample	366/1632	56.8	71.3	16.3	CRAFFT substance abuse screening test	Alcohol	25.1/11.0
Ko et al. (2008) [23]	Taiwan	Case -control	Respondents to an advertisement regarding internet usage	DC-IA-C	Community sample	87/129	61.1	74.7	21.5	Chinese Version of the Mini-International Neuropsychiatric interview	Depression	18.3/6.2
											ADHD	32.2/8.5
											Anxiety	14.9/6.2
Yoo et al. (2004) [24]	Korea	Case- control	Elementary school students	IAT	Community sample	80/455	49.3	68.8	11.1	K-ARS	ADHD	22.5/8.1

YDQ, Young diagnostic questionnaire; IAT , Young Internet Addiction test; CIAS Chen Internet Addiction Scale; DC-IA-C, Diagnostic Criteria of Internet Addiction for College; SCL-90-R , Symptom Checklist-90-Revision; CES-D, Center of Epidemiologic Studies Depression Scale; ADHDS, Attention-Deficit/hyperactivity Disorder Self-rated Scale; BV-FNE, Brief Version of the Fear of Negative Evaluation Scale; AUDIT, Alcohol Use Disorders Identification Test; ASRS, Adult ADHD Self-Report Scale; K-ARS, Korean version of DuPaul’s ADHD rating scale.

**Table 2:** Results of subgroup effect size analysis of internet addiction patients for each of the psychiatric comorbidity

**Subgroups**	**No. of studies**	**Pooled prevalence (%)**	**95% CI**	**P value in between-group comparison**
1. Attention deficit and hyperactivity				
Young adults (19-39 years)	2	23.7	17.0-32.0	<0.001
Adolescents (10-18 years)	2	20.3	16.3-24.9	
2. Alcohol abuse				
Middle-aged adults and elderly (40-76 years)	1	6.1	3.6-10.2	<0.001
Young adults (19-39 years)	1	13.0	9.3-17.8	
Adolescents (10-18 years)	1	25.1	20.9-29.8	
3. Anxiety				
Middle-aged adults and elderly (40-76 years)	1	16.5	12.1-22.1	<0.001
Young adults (19-39 years)	2	30.9	8.6-68.1	
Adolescents (10-18 years)	1	20.3	15.9-25.6	
4. Depression				
Middle-aged adults and elderly (40-76 years)	1	35.8	29.7-42.5	<0.001
Young adults (19-39 years)	3	28.3	18.2-41.3	
Adolescents (10-18 years)	1	14.3	10.5-19.2	

### Alcohol abuse

The prevalence of alcohol abuse among IA patients was 13.3% (95% CI: 5.9% - 27.0%, z = -4.158, df = 2, τ^2^ = 0.57, I^2^ = 94.18). The prevalence of alcohol abuse among the normal controls was 4.3% (95% CI: 1.4%–12.2%, z = -5.370, df = 2, τ^2^ = 0.99, I^2^ = 98.8). Figure 1 demonstrates the results of three studies which compared the prevalence of alcohol abuse between patients with IA and their healthy counterparts. The proportion of patients with alcohol abuse was significantly higher in the IA group than in the control group (pooled OR = 3.05, 95% CI: 2.14-4.37, z = 6.12, P < 0.001). There was a moderate but non-significant degree of between-study heterogeneity (τ^2^ = 0.052; Q = 4.22, df = 2, P = 0.121, I^2^ = 52.6).

### Attention deficit and hyperactivity disorder

The prevalence of ADHD among IA patients was 21.7% (95% CI: 18.6 – 25.0%, z = -13.283, df = 3, τ^2^ = 0.005, I^2^ = 12.4). The prevalence of ADHD among the normal controls was 8.9% (95% CI: 7.9–10.1%, z = -35.0, df = 3, τ^2^ = 0.004, I^2^ = 22.59). Figure 2 demonstrates the results of four studies which compared the prevalence of ADHD between patients with IA and controls. The proportion of patients with ADHD was significantly higher in the IA group than in the control group (pooled OR 2.85, 95% CI: 2.15-3.77, z = 7.27, P < 0.001). There was a low and non-significant level of between-study heterogeneity (τ^2^ = 0.029; Q = 4.68, df = 3, P = 0.197, I^2^ = 35.9).

### Depression

The prevalence of depression among IA patients was 26.3% (95% CI: 17.6–37.4%, z = -3.93, df =4, τ^2^ = 0.30, I^2^ = 88.99). The prevalence of depression among the normal controls was 11.7% (95% CI: 8.8–15.5%, Q = 10.205, df = 4, p < 0.001, τ^2^ = 0.11, I^2^ = 92.57). Figure 3 demonstrates the results of five studies which compared the prevalence of depression between patients suffering from IA and the healthy controls. The proportion of patients with depression was significantly higher in the IA group than in the control group (pooled OR = 2.77, 95% CI = 2.04–3.75, z = 6.55, P < 0.001) based on the random-effects model. The between-study heterogeneity was moderate but not statistically significant (τ^2^ = 0.062, Q = 9.1, df = 4, P = 0.06, I^2^ = 56.1).

### Anxiety

The prevalence of anxiety among IA patients was 23.3% (95% CI: 14.8-34.8, z = - 4.156, df = 3, τ^2^ = 0.27, I^2^ = 84.37). The prevalence of anxiety among the normal controls was 10.3% (95% CI: 5.0–19.9, z = -5.47, df = 3, τ^2^ = 0.59, I^2^ = 97.9). Figure 4 demonstrates the result of four studies which compared the prevalence of anxiety symptoms between patients with IA and controls. The proportion of patients with anxiety symptoms was significantly higher in the IA group than in the control group (pooled OR = 2.70, 95% CI: 1.46-4.97, z = 3.18, P = 0.001). There was a high level of between-study heterogeneity (τ^2^ = 0.293; Q = 16.0, df = 3, P = 0.001, I^2^ = 81.2).

### Subgroup analysis

The pattern and aggregate prevalence of psychiatric co-morbidity were significantly different across the three age-stratified subgroups (Table 2). ADHD was more prevalent in studies involving young adults (19-39 years of age). Amongst IA patients, anxiety was the most prevalent among young adults (19-39 years of age); depression was the most prevalent among middle-aged adults and the elderly (40-76 years); alcohol abuse was the most prevalent among adolescents (10- 18 years of age).

### Publication bias

There was no significant publication bias for any of the psychiatric co-morbidity: alcohol abuse (intercept = 2.86, 95% CI: -28.9-34.6, τ = 1.14, df = 1, P = 0.458), ADHD (intercept = 2.19, 95% CI: 4.63-9.10, τ^2^ = 1.38, df = 2, P = 0.3), depression (intercept = 0.29, 95% CI: -6.42-6.99, τ^2^ = 0.14 df = 3, P = 0.90) and anxiety (intercept = 3.24, 95% CI: -9.26-15.7, τ^2^ = 1.12; df =2, P = 0.38).

## Discussion

To our knowledge, this is the first meta-analysis to investigate the relationship between IA and psychiatric co-morbidity with consideration of heterogeneity. Our findings suggest that IA is associated with alcohol abuse, ADHD, depression and anxiety. Amongst these significant psychiatric co-morbidities, alcohol abuse has the strongest association with IA. Carli et al. [8] reported that 75% of pathological internet users suffer from depression, 57% from anxiety and 100% from ADHD. In the present study, 26.3% of patients with IA suffer from depression, 23.3% from anxiety and 21.7% from ADHD. Our findings are similar to the prevalence of coexisting psychiatric disorders in patients suffering from alcohol and drug disorders which ranges between 20% and 30% [25]. Resultant findings support our hypothesis that the prevalence of psychiatric co-morbidity in IA is similar to that in substance use and addictive disorders. A previous study by Carli et al. may have overestimated the prevalence of psychiatric co-morbidity in IA, especially that of ADHD. Contrary to alcohol abuse, depression and ADHD, the aggregate prevalence of anxiety demonstrates significant heterogeneity. Subgroup analysis showed that the aggregate prevalence of the respective psychiatric symptoms varied significantly based on the age stratification of the sample.

Our results suggest that IA and psychiatric co-morbidity may co-occur as a result of complex interaction between various aetiological factors. The intricacies of the genetic transmission of IA remains exploratory. Montag et al. [26] found that the CC genotype of the rs1044396 polymorphism on gene coding for the nicotinic acetylcholine receptor subunit alpha 4 (CHRNA4) occurred significantly more frequently in patients suffering from IA. Moreover, nicotinic receptors play a key role in nicotine addiction. Lee et al. [27] reported that the homozygous short alleles (SS) of the serotonin transporter gene promoter region (5HTTLPR) are more prevalent among excessive internet users, and the genotype was found to be associated with depressive disorder [28]. The involvement of the serotonin genotype in IA and depression suggests that these two conditions may share similar neurochemical changes thereby warranting further investigation.

Patients suffering from IA are more likely to be non-compliant with psychotropic medication and psychotherapy because they are preoccupied with internet usage. Furthermore, depression and anxiety often occur as part of the internet withdrawal syndrome. Excessive internet usage may serve as a maintaining factor for anxiety by reinforcing the avoidance of anticipatory anxiety stemming from stressful situations and life events. The relationship between IA and alcohol is complicated. The biological reinforcement models [25] suggest that alcohol may alter neuroanatomical pathways that are involved in the positive reinforcement of internet use. Although internet-based treatment has been used for detrimental alcohol use [29], there is a paucity of research in the effects of alcohol on internet use. Wu and Delva [30] reported that the use of internet at home has no effect on drinking but the use of computers in internet cafés was a strong predictor of drinking among women. Yen et al. reported that fun-seeking is a shared characteristic of IA and alcohol abuse [19]. The above-mentioned findings support our subgroup analysis which demonstrated that alcohol abuse is more prevalent among younger subjects (10-18 years of age), as internet cafés and fun-seeking are typically more common among youths. While it is unlikely that IA causes ADHD, clinical impressions posit that internet usage may improve ADHD. In our subgroup analysis, ADHD is more prevalent in young adults (19-39 years of age). Clinical observations point to a predominance of inattention over and above hyperactivity in adults. The self-treatment hypothesis thus postulates that adult patients with ADHD use the internet excessively to control their inattention. Furthermore, the onset of ADHD (at age 7 years by clinical definition) usually predates the incipience of IA [31], thereby suggesting the potential role of ADHD as a predisposing factor of IA.

Present emergent findings confer significant clinical implications as they aid mental health professionals in appreciating that IA may not present as a singular diagnostic entity, but co-occur with alcohol abuse, ADHD, depression and anxiety. It is thus reasonable to expect that all patients who present with IA be adequately screened for alcohol abuse, ADHD, depression and anxiety. In a similar vein, it is recommended that patients who present with the above-mentioned psychiatric condition be interviewed about their internet usage. Our findings also inform the prospective treatment of IA because psychiatric co-morbidity may reinforce, if not maintain the pathological pattern of internet usage. Therefore, the treatment of IA and psychiatric co-morbidity should be integrated into a cohesive service which focuses on the minimization of harm [25]. The overarching goals of treatment would encompass the abstinence of internet usage, activity scheduling to supplant online activity with face-to-face interpersonal activity, and motivational interviewing. The reduction of internet usage may lead to a corresponding reduction of the severity of comorbid psychiatric symptoms. Pharmacotherapy involving the opioid receptor antagonist, naltrexone may diminish euphoria or the rewarding experience that is associated with alcohol abuse and IA. Similarly, selective serotonin reuptake inhibitors (SSRIs) may attenuate the impulsivity that is associated with internet use, depression and anxiety. For patients who tend to use the internet in internet cafés which serve alcoholic beverages, avoidance of those locations or short-term hospitalisation may treat both conditions simultaneously.

A strength of this meta-analysis is the minimal publication bias. Notwithstanding, this meta-analysis is not without its limitations. Firstly, we could not assess cause-and-effect mechanisms underpinning IA and psychiatric co-morbidity because most studies included are cross-sectional in nature. Secondly, the association between IA and psychiatric co-morbidity should be interpreted with caution because there exist confounding factors, for example attachment [32], environmental stress, parenting styles [33] family structure [34], and gender. In extant case-control studies [19,20,24], the proportion of males is significantly higher in the IA groups as compared to control groups. ADHD and alcohol abuse are also known to be more common in males than females [35]. Furthermore, the number of studies focusing on alcohol abuse is small (n = 3 studies). Thirdly, studies focusing on other psychiatric co-morbidities such as eating disorders and abuse of other recreational drugs did not meet the inclusion criteria and were not included. Although there exist other studies which reported the severity levels of depression, anxiety and ADHD, these studies were excluded because different questionnaires were used and the scores could not be combined. Most studies included in the meta-analysis employed self-report questionnaires with the exception of a single study which had established the diagnosis of psychiatric co-morbidity using a structured interview format [23]. Due to the small number of studies, we could not perform a sensitivity analysis to investigate the differences in psychiatric co-morbidity that was established by self-reported questionnaires and structured interviews as well as the differences in pooled OR between varying study designs. The meta-analysis was also limited in that the scales and structured interviews administered focused primarily on IA but not IA-specific behaviours such as gaming, shopping and social media. Consequently, we could not establish the psychiatric co-morbidity associated with specific types of IA behaviour. A fifth limitation resides in a high and statistically significant level of between-study heterogeneity that was found in the pooled OR for anxiety. This warrants further meta-regression to identify moderators that may be attributing to the significant heterogeneity. Meta-regression was not performed in this study because Gagnier et al. has recommended at least 10 studies per moderator in meta-regression to avoid spurious findings. Finally, most of the subjects included in this meta-analysis were young Asians from China and Korea. Further studies are required to investigate other ethnic groups in Europe and North America, as well as older adults. It is important to note that the patterns of internet use and recreational drug use may vary between Eastern and Western populations.

Further research is necessitated to arrive at a consensus on the definition of IA and examine the unique interactions between IA and psychiatric co-morbidity such as common aetiology, illness trajectory and treatment outcomes. In this meta-analysis, the definition of IA was based on two instruments, namely Young’s Internet Addiction Test and Chen Internet Addiction Scale. Although there are overlapping characteristics between two questionnaires, further research is required to arrive at a consensus on the diagnostic criteria of IA.

With the exception of ADHD (age of onset is known to be earlier than 7 years) [31], the sequence of development of IA and other psychiatric co-morbidity remains unclear. Prospective studies are required to determine if psychiatric co-morbidities such as depression and anxiety originate from IA and abate with reduced usage of internet or otherwise. Also, as patients suffering from IA and psychiatric co-morbidity may not respond to standard treatment approaches, further research is required to investigate the effect of psychotropic medications (e.g. antidepressant, methylphenidate) on the severity of IA and psychiatric co-morbidity.

## Conclusion

The present meta-analysis engendered a significant association between IA and alcohol abuse, ADHD, depression and anxiety. Psychiatric co-morbidity occurs in a range from 13.3% to 26.3% among patients suffering from IA. To enhance understanding of the relationship between IA and psychiatric co-morbidity, prospective studies involving other psychiatric co-morbidities are required to establish the cause-and-effect mechanisms between IA and psychiatric co-morbidity. In view that the complex interactions between IA and psychiatric co-morbidity may lead to significant health burden, concerted mental health services need to be developed in anticipation of the recognition of IA as a diagnosable psychiatric disorder in the future.

## Competing interests

The authors declared that they have no competing interests.

## Authors’ contributions

RCH contributed to conception and design, analysis and interpretation of data, drafting the article and revising it critically for important intellectual content, and final approval of the version to be published. MWZ contributed to analysis and interpretation of data, and drafting the article. TYT contributed to drafting the article and interpretation of data. AHT, FP, YL, CC, PSY, LTL, CML, HW revised the article critically for important intellectual content. KKM contributed to conception and design, analysis and interpretation of data, revising the article critically for important intellectual content, and final approval of the version to be published. All authors read and approved the final manuscript.

## Pre-publication history

The pre-publication history for this paper can be accessed here:

http://www.biomedcentral.com/1471-244X/14/183/prepub

## Supplementary Material

Additional file 1: Table S1 Summarizes all studies published in Chinese language and the reasons for exclusion. Click here for file

Additional file 2: Table S2 Summarizes all studies published in Korean language and the reasons for exclusion. Click here for file

## References

[B1] Block JJMD Issues for DSM-V: internet addiction Am J Psychiatry 2008 165 3 306 307 18316427 10.1176/appi.ajp.2007.07101556

[B2] American Psychiatric Association Diagnostic and Statistical Manual of Mental Disorders DSM-5 2013 5 Washington: American Psychiatric Publishing

[B3] Ko CH Yen JY Yen CF Chen CS Chen CC The association between Internet addiction and psychiatric disorder: a review of the literature Eur Psychiatry 2012 27 1 1 8 22153731 10.1016/j.eurpsy.2010.04.011

[B4] Weinstein A Lejoyeux M Internet addiction or excessive internet use Am J Drug Alcohol Abuse 2010 36 5 277 283 20545603 10.3109/00952990.2010.491880

[B5] Morrison CM Gore H The relationship between excessive Internet use and depression: a questionnaire-based study of 1,319 young people and adults Psychopathology 2010 43 2 121 126 20110764 10.1159/000277001

[B6] Cheung LM Wong WS The effects of insomnia and internet addiction on depression in Hong Kong Chinese adolescents: an exploratory cross-sectional analysis J Sleep Res 2011 20 2 311 317 20819144 10.1111/j.1365-2869.2010.00883.x

[B7] Yen JY Ko CH Yen CF Wu HY Yang MJ The comorbid psychiatric symptoms of Internet addiction: attention deficit and hyperactivity disorder (ADHD), depression, social phobia, and hostility J Adolesc Health 2007 41 1 93 98 17577539 10.1016/j.jadohealth.2007.02.002

[B8] Carli V Durkee T Wasserman D Hadlaczky G Despalins R Kramarz E Wasserman C Sarchiapone M Hoven CW Brunner R Kaess M The association between pathological internet use and comorbid psychopathology: a systematic review Psychopathology 2011 46 1 1 13 22854219 10.1159/000337971

[B9] Lai CM Mak KK Watanabe H Ang RP Pang JS Ho RC Psychometric properties of the internet addiction test in Chinese adolescents J Pediatr Psychol 2013 38 7 794 807 23671059 10.1093/jpepsy/jst022

[B10] Mak KK Lai CM Ko CH Chou C Kim DI Watanabe H Ho RC Psychometric properties of the revised chen internet addiction scale (CIAS-R) in Chinese adolescents J Abnorm Child Psychol 2014 [Epub ahead of print] 10.1007/s10802-014-9851-3 24585392

[B11] Stroup DF Berlin JA Morton SC Olkin I Williamson GD Rennie D Moher D Becker BJ Sipe TA Thacker SB Meta-analysis of observational studies in epidemiology: a proposal for reporting. meta-analysis Of observational studies in epidemiology (MOOSE) group JAMA 2000 283 15 2008 2012 10789670 10.1001/jama.283.15.2008

[B12] Han B Eskin E Random-effects model aimed at discovering associations in meta-analysis of genome-wide association studies Am J Hum Genet 2011 88 5 586 598 21565292 10.1016/j.ajhg.2011.04.014 PMC3146723

[B13] Diggle P Analysis of longitudinal data 2002 2 Oxford, New York: Oxford University Press

[B14] Fitzmaurice GM Laird NM Ware JH Applied longitudinal analysis 2004 Hoboken, N.J.: Wiley-Interscience

[B15] Laird NM Ware JH Random-effects models for longitudinal data Biometrics 1982 38 4 963 974 7168798

[B16] Bridle C Spanjers K Patel S Atherton NM Lamb SE Effect of exercise on depression severity in older people: systematic review and meta-analysis of randomised controlled trials Br J Psychiatry 2012 201 3 180 185 22945926 10.1192/bjp.bp.111.095174

[B17] Alavi SS Maracy MR Jannatifard F Eslami M The effect of psychiatric symptoms on the internet addiction disorder in Isfahan's University students J Res Med Sci 2011 16 6 793 800 22091309 PMC3214398

[B18] Ko CH Yen JY Chen CS Yeh YC Yen CF Predictive values of psychiatric symptoms for internet addiction in adolescents: a 2-year prospective study Arch Pediatr Adolesc Med 2009 163 10 937 943 19805713 10.1001/archpediatrics.2009.159

[B19] Yen JY Ko CH Yen CF Chen CS Chen CC The association between harmful alcohol use and Internet addiction among college students: comparison of personality Psychiatry Clin Neurosci 2009 63 2 218 224 19335391 10.1111/j.1440-1819.2009.01943.x

[B20] Yen JY Yen CF Chen CS Tang TC Ko CH The association between adult ADHD symptoms and internet addiction among college students: the gender difference Cyberpsychol Behav 2009 12 2 187 191 19072077 10.1089/cpb.2008.0113

[B21] Bakken IJ Wenzel HG Gotestam KG Johansson A Oren A Internet addiction among Norwegian adults: a stratified probability sample study Scand J Psychol 2009 50 2 121 127 18826420 10.1111/j.1467-9450.2008.00685.x

[B22] Ko CH Yen JY Yen CF Chen CS Weng CC Chen CC The association between Internet addiction and problematic alcohol use in adolescents: the problem behavior model Cyberpsychol Behav 2008 11 5 571 576 18785835 10.1089/cpb.2007.0199

[B23] Ko CH Yen JY Chen CS Chen CC Yen CF Psychiatric comorbidity of internet addiction in college students: an interview study CNS Spectr 2008 13 2 147 153 18227746 10.1017/s1092852900016308

[B24] Yoo HJ Cho SC Ha J Yune SK Kim SJ Hwang J Chung A Sung YH Lyoo IK Attention deficit hyperactivity symptoms and internet addiction Psychiatry Clin Neurosci 2004 58 5 487 494 15482579 10.1111/j.1440-1819.2004.01290.x

[B25] Todd F Hulse G, White JM, Cape G Coexisting alcohol and drug use and mental heath disorders Management of alcohol and drug problems 2004 South Melbourne, Vic., Australia: Oxford University Press 404

[B26] Montag C Kirsch P Sauer C Markett S Reuter M The role of the CHRNA4 gene in Internet addiction: a case-control study J Addict Med 2012 6 3 191 195 22722381 10.1097/ADM.0b013e31825ba7e7

[B27] Lee YS Han DH Yang KC Daniels MA Na C Kee BS Renshaw PF Depression like characteristics of 5HTTLPR polymorphism and temperament in excessive internet users J Affect Disord 2008 109 1–2 165 169 18045695 10.1016/j.jad.2007.10.020

[B28] Mak KK Kong WY Mak A Sharma VK Ho RC Polymorphisms of the serotonin transporter gene and post-stroke depression: a meta-analysis J Neurol Neurosurg Psychiatry 2012 84 3 322 8 23236014 10.1136/jnnp-2012-303791

[B29] Blankers M Nabitz U Smit F Koeter MW Schippers GM Economic evaluation of internet-based interventions for harmful alcohol use alongside a pragmatic randomized controlled trial J Med Internet Res 2012 14 5 e134 23103771 10.2196/jmir.2052 PMC3517375

[B30] Wu L Delva J The effect of computer usage in internet cafe on cigarette smoking and alcohol use among chinese adolescents and youth: a longitudinal study Int J Environ Res Public Health 2012 9 2 496 510 22470305 10.3390/ijerph9020496 PMC3315259

[B31] American Psychiatric Association., American Psychiatric Association. Task Force on DSM-IV Diagnostic and statistical manual of mental disorders: DSM-IV-TR 2000 4 Washington, DC: American Psychiatric Association

[B32] Shin SE Kim NS Jang EY Comparison of problematic internet and alcohol use and attachment styles among industrial workers in Korea Cyberpsychol Behav Soc Netw 2011 14 11 665 672 21595524 10.1089/cyber.2010.0470

[B33] Xiuqin H Huimin Z Mengchen L Jinan W Ying Z Ran T Mental health, personality, and parental rearing styles of adolescents with Internet addiction disorder Cyberpsychol Behav Soc Netw 2010 13 4 401 406 20712498 10.1089/cyber.2009.0222

[B34] Ni X Yan H Chen S Liu Z Factors influencing internet addiction in a sample of freshmen university students in China Cyberpsychol Behav 2009 12 3 327 330 19445631 10.1089/cpb.2008.0321

[B35] Puri B, Hall A, Ho RC Revision Notes in Psychiatry 2014 third Oxford

