# Supplementary material for: The association between internet addiction and psychiatric co-morbidity: a meta-analysis
Source: BMC Psychiatry. 2014 Jun 20;14:183. doi: 10.1186/1471-244X-14-183 (PMC4082374; doi:10.1186/1471-244X-14-183)
Supplement: Additional file 1: Table S1 — Summarizes all studies published in Chinese language and the reasons for exclusion. [file 1471-244X-14-183-S1.docx]

**Additional file 1 Table S1 summarizes all studies published in Chinese language and the reasons for exclusion.**

| **Studies** | **Reasons for exclusions** |
| --- | --- |
| [A voxel-based morphometric analysis of brain gray matter in online game addicts.](http://www.ncbi.nlm.nih.gov/pubmed/23328472) Weng CB, Qian RB, Fu XM, Lin B, Ji XB, Niu CS, Wang YH. Zhonghua Yi Xue Za Zhi. 2012 Dec 4;92(45):3221-3. Chinese. | This is an imaging study and did not report psychiatric co-morbidity. |
| [Relationship of childhood physical abuse and internet addiction disorder in adolescence: the mediating role of self-esteem.](http://www.ncbi.nlm.nih.gov/pubmed/22575110)  Zhang ZH, Yang LS, Hao JH, Huang F, Zhang XJ, Sun YH.  Zhonghua Liu Xing Bing Xue Za Zhi. 2012 Jan;33(1):50-3. Chinese. | This study focused on abuse and did not report psychiatric co-morbidity. |
| [Intervention on network craving and encephalofluctuogram in patients with internet addiction disorder: a randomized controlled trial.](http://www.ncbi.nlm.nih.gov/pubmed/21692281) Zhu TM, Li H, Du YP, Zheng Z, Jin RJ. Zhongguo Zhen Jiu. 2011 May;31(5):395-9. Chinese. | This is an EEG study. |
| [Influence of excessive internet use on auditory event-related potential.](http://www.ncbi.nlm.nih.gov/pubmed/19166194)  Zhao X, Yu H, Zhan Q, Wang M. Sheng Wu Yi Xue Gong Cheng Xue Za Zhi. 2008 Dec;25(6):1289-93. Chinese.  [The effect of excessive internet use on N400 event-related potentials.](http://www.ncbi.nlm.nih.gov/pubmed/19024437)  Yu H, Zhao X, Wang Y, Li N, Wang M.  Sheng Wu Yi Xue Gong Cheng Xue Za Zhi. 2008 Oct;25(5):1014-20. Chinese. | These studies focused on event-related potentials. |
| [Analysis on the epidemiology of 607 inpatients with internet addiction disorder.](http://www.ncbi.nlm.nih.gov/pubmed/17877193) Tao R, Huang XQ, Yao SM.  Zhonghua Liu Xing Bing Xue Za Zhi. 2007 May;28(5):519. Chinese.  [Analysis on the epidemiology of 607 inpatients with internet addiction disorder.](http://www.ncbi.nlm.nih.gov/pubmed/17877193) Tao R, Huang XQ, Yao SM.  Zhonghua Liu Xing Bing Xue Za Zhi. 2007 May;28(5):519. Chinese.  [Internet addiction among Shanghai adolescents: prevalence and epidemiological features.](http://www.ncbi.nlm.nih.gov/pubmed/19178835) Xu J, Shen LX, Yan CH, Wu ZQ, Ma ZZ, Jin XM, Shen XM. Zhonghua Yu Fang Yi Xue Za Zhi. 2008 Oct;42(10):735-8. Chinese. | These studies reported prevalence of IA but not psychiatric co-morbidity. |
| [Status of 'internet addiction disorder' (IAD) and its risk factors among first-grade junior students in Wuhan.](http://www.ncbi.nlm.nih.gov/pubmed/20302690) Song XQ, Zheng L, Li Y, Yu DX, Wang ZZ.  Zhonghua Liu Xing Bing Xue Za Zhi. 2010 Jan;31(1):14-7. Chinese | This study reported risk factors leading to IA but not psychiatric co-morbidity. |
| [Clinical effect of electroacupuncture combined with psychologic interference on patient with Internet addiction disorder.](http://www.ncbi.nlm.nih.gov/pubmed/19548435) Zhu TM, Jin RJ, Zhong XM. Zhongguo Zhong Xi Yi Jie He Za Zhi. 2009 Mar;29(3):212-4. Chinese.  [Effects of electroacupuncture combined with psychologic interference on anxiety state and serum NE content in the patient of internet addiction disorder.](http://www.ncbi.nlm.nih.gov/pubmed/18767577) Zhu TM, Jin RJ, Zhong XM, Chen J, Li H. Zhongguo Zhen Jiu. 2008 Aug;28(8):561-4. Chinese. | These studies focused on acupuncture. |
| [Parental rearing styles and personality characteristics of young inpatients with internet addiction disorders in different family types.](http://www.ncbi.nlm.nih.gov/pubmed/18396679) Tao R, Huang XQ, Zhang HM. Zhonghua Liu Xing Bing Xue Za Zhi. 2007 Nov;28(11):1153-4. Chinese | This study reported personality but not psychiatric co-morbidity. |
| [Association between adolescent internet addiction and suicidal behaviors.](http://www.ncbi.nlm.nih.gov/pubmed/21162812)  Yang LS, Zhang ZH, Hao JH, Sun YH. Zhonghua Liu Xing Bing Xue Za Zhi. 2010 Oct;31(10):1115-9. Chinese. | This study reported suicidal ideation and impulsivity but not psychiatric co-morbidity. |
