# Supplementary material for: The association between internet addiction and psychiatric co-morbidity: a meta-analysis
Source: BMC Psychiatry. 2014 Jun 20;14:183. doi: 10.1186/1471-244X-14-183 (PMC4082374; doi:10.1186/1471-244X-14-183)
Supplement: Additional file 2: Table S2 — Summarizes all studies published in Korean language and the reasons for exclusion. [file 1471-244X-14-183-S2.docx]

**Additional file 2 Table S2 summarizes all studies published in Korean language and the reasons for exclusion.**

| [Effects of a group counseling integration program on self-determination and Internet addiction in high school students with tendency to Internet addiction.](http://www.ncbi.nlm.nih.gov/pubmed/22143218)  Park GR, Kim HS. J Korean Acad Nurs. 2011 Oct;41(5):694-703. doi: 10.4040/jkan.2011.41.5.694. Korean. | This study focused on counselling but not psychiatric co-morbidity. |
| --- | --- |
| [A prediction model for internet game addiction in adolescents: using a decision tree analysis.](http://www.ncbi.nlm.nih.gov/pubmed/20634629) Kim KS, Kim KH. J Korean Acad Nurs. 2010 Jun;40(3):378-88. doi: 10.4040/jkan.2010.40.3.378. Korean. | This study focused on decision making process but not psychiatric co-morbidity. |
| [Effects of an empowerment education program in the prevention of internet games addiction in middle school students.](http://www.ncbi.nlm.nih.gov/pubmed/20453572) Joo A, Park I.  J Korean Acad Nurs. 2010 Apr;40(2):255-63. doi: 10.4040/jkan.2010.40.2.255. Korean. | This study focused on education program but not psychiatric co-morbidity. |
| [Relationships among cybersex addiction, gender egalitarianism, sexual attitude and the allowance of sexual violence in adolescents.](http://www.ncbi.nlm.nih.gov/pubmed/18182882) Koo HY, Kim SS.  Taehan Kanho Hakhoe Chi. 2007 Dec;37(7):1202-11. Korean. | This study focused on sexual attitudes and sexual violence but not psychiatric co-morbidity. |
| A study of factors that influence Internet addiction, smoking, and drinking in high school students. [June KJ](http://www.ncbi.nlm.nih.gov/pubmed?term=June%20KJ%5BAuthor%5D&cauthor=true&cauthor_uid=17992059), [Sohn SY](http://www.ncbi.nlm.nih.gov/pubmed?term=Sohn%20SY%5BAuthor%5D&cauthor=true&cauthor_uid=17992059), [So AY](http://www.ncbi.nlm.nih.gov/pubmed?term=So%20AY%5BAuthor%5D&cauthor=true&cauthor_uid=17992059), [Yi GM](http://www.ncbi.nlm.nih.gov/pubmed?term=Yi%20GM%5BAuthor%5D&cauthor=true&cauthor_uid=17992059), [Park SH](http://www.ncbi.nlm.nih.gov/pubmed?term=Park%20SH%5BAuthor%5D&cauthor=true&cauthor_uid=17992059).  [Taehan Kanho Hakhoe Chi.](http://www.ncbi.nlm.nih.gov/pubmed/17992059) 2007 Oct;37(6):872-82. | In this study, the percentages of subjects who drank alcohol once or twice per month were recorded. The frequency of drinking was too low and not considered to be alcohol misuse. |
| [Factors on internet game addiction among adolescents.](http://www.ncbi.nlm.nih.gov/pubmed/17804942)  Park HS, Kwon YH, Park KM. Taehan Kanho Hakhoe Chi. 2007 Aug;37(5):754-61. Korean. | This study did not report psychiatric co-morbidity. |
| [Development of internet addiction measurement scales and Korean internet addiction index.](http://www.ncbi.nlm.nih.gov/pubmed/16323630) Park JS. J Prev Med Public Health. 2005 Aug;38(3):298-306. Korean. | This study reported a new scale to assess internet addiction but did not report prevalence of psychiatric co-morbidity. |
| [Association of Internet addiction with health promotion lifestyle profile and perceived health status in adolescents.](http://www.ncbi.nlm.nih.gov/pubmed/16312910) Kim JS, Chun BC.  J Prev Med Public Health. 2005 Feb;38(1):53-60. Korean. | This study focused on lifestyle but not psychiatric co-morbidity. |
| [The relationships of Internet addiction, depression, and suicidal ideation in adolescents.](http://www.ncbi.nlm.nih.gov/pubmed/15314344) Ryu EJ, Choi KS, Seo JS, Nam BW. Taehan Kanho Hakhoe Chi. 2004 Feb;34(1):102-10. Korean. | This study reported the mean depression scores. This study did not report the number of subjects suffering from each psychiatric co-morbidity. |
| [Immersion experience of the cyber world of adolescents.](http://www.ncbi.nlm.nih.gov/pubmed/15314335)  Park NH, Cho YR, Choi WH, Moon NJ, An HG, Shin JS.  Taehan Kanho Hakhoe Chi. 2004 Feb;34(1):15-24. Korean. | This study did not report psychiatric co-morbidity. |
| [A study on a model for internet addiction of adolescents.](http://www.ncbi.nlm.nih.gov/pubmed/15314311) Cho YR, Lee HJ.  Taehan Kanho Hakhoe Chi. 2004 Jun;34(3):541-51. Korean. | This study did not report psychiatric co-morbidity. |
